# The importance of magnetic resonance imaging in the differential diagnosis of spinal cord injuries

**DOI:** 10.1590/0100-3984.2021.54.6e1

**Published:** 2021

**Authors:** Alair Augusto Sarmet M. D. dos Santos

**Affiliations:** 1 Professor Associado/Chefe do Departamento de Radiologia da Faculdade de Medicina da Universidade Federal Fluminense (UFF), Coordenador Médico do Centro de Imagens do Complexo Hospitalar de Niterói (CHN), Niterói, RJ, Brasil.

Several types of injuries can affect the spinal cord, with similar clinical presentations, including motor, sensory, and autonomic impairment. Magnetic resonance imaging (MRI) is the fundamental imaging method for the investigation of such injuries and the planning of their appropriate treatment. In many cases, the final diagnosis is delayed because MRI is not performed^(^1-6^)^. The various causes of spinal cord injury include neoplasms (e.g., astrocytomas and, more frequently, ependymomas), trauma, vascular diseases, infectious diseases, inflammatory diseases, and metabolic disorders.

This issue of Radiologia Brasileira presents two companion articles dealing with spinal cord injuries^(^7,8^)^. The authors describe the clinical presentation and the various MRI aspects of such injuries. When performing MRI examinations of patients with spinal cord injuries, it is important to pay attention to the correct identification of signal changes in the spinal cord and always to use paramagnetic contrast, which significantly improves the delineation of the lesions, the demonstration of activity (or inactivity) within the lesion (in patients with inflammatory diseases), and the visualization of the lesions. It is also important to acquire images in the coronal plane, in addition to the usual acquisitions in the sagittal and axial planes, which are routinely performed at most centers^(^1-3^)^. The two articles briefly present the different types of spinal cord lesions, highlighting the importance of considering sex, age, and clinical profile in order to reach the most appropriate diagnosis.

Although astrocytoma and ependymoma are the most common neoplastic causes of spinal cord injury, they account for only 2-3% of primary intra-axial central nervous system tumors in the spinal cord. Ependymomas occur predominantly in adults, whereas astrocytomas occur predominantly in children. On MRI, spinal cord edema and contrast enhancement facilitate the differential diagnosis between astrocytomas and ependymomas. Ganglioglioma, hemangioblastoma, and spinal cord metastases are rare, the most common primary sites of metastases being lung and breast tumors, which usually spread through hematogenous dissemination^(^3-6^)^.

One of the main metabolic causes of spinal cord injury is vitamin B12 deficiency, which can manifest as degeneration of the bone marrow and has become a major consideration in Brazil, due to the great increase in the number of people who have undergone bariatric surgery, in whom this deficiency occurs more often. Knowledge of the pathological history is of paramount importance, because spinal cord changes may regress with adequate vitamin B12 replacement through monthly intramuscular injections^(^9^)^.

A common cause of acute myelopathy is spinal cord trauma, whether due to motor vehicle accidents, falls, or accidents occurring in swimming pools or other bodies of water. Although such trauma is often accompanied by changes in the osseous framework of the spine, it is important to be aware of bone marrow involvement, which is not well evaluated by computed tomography alone, MRI being essential to demonstrate edema, contusion, and spinal cord hemorrhage, because it is more accurate than is tomography in assessing spinal cord involvement^(^10^)^.

Among the vascular diseases affecting the spinal cord, special attention should be given to spinal cavernous malformations, which are slightly more common in women and in the fourth decade of life, typically being seen in the thoracic spine, with an appearance similar to that of intracranial cavernous malformations, without contrast enhancement^(^6^)^.

In patients with multiple sclerosis, it is necessary to look for spinal cord lesions, because the presence of active plaques is indicative of the activity of the underlying disease. It is important to make the differential diagnosis with other inflammatory diseases, such as acute disseminated encephalomyelitis, neuromyelitis optica, transverse myelitis, myelin oligodendrocyte glycoprotein antibody-associated myelitis, and systemic lupus erythematosus, which is, in rare cases, accompanied by myelitis in the thoracic spine^(^1,2,6^)^.

In Brazil, spinal cord lesions of infectious causes have great repercussions, because they are often diagnosed late, given the limited access to MRI examinations, especially in the public health care system. The viral agents that can cause spinal cord lesions include the Zika virus, human T-lymphotropic virus I, HIV, and the varicella-zoster virus. In Guillain-Barré syndrome caused by Zika virus infection, there can be polyneuropathy with acute demyelination and involvement of the conus medullaris^(^11^)^. Spinal cord tuberculosis is a rare entity that can progress to ischemia and bone marrow necrosis. Schistosomiasis is another disease that can lead to acute/subacute myelopathy, with greater expression in the conus medullaris, together with thickening of and enhancement in the roots of the cauda equina.

In patients with spinal cord injury, it is essential for physicians, especially neurologists and neurosurgeons, to be aware of the various diagnostic possibilities, as well as for radiologists to be aware of the various aspects seen on MRI, in order to elucidate the diagnosis as early as possible. The choice between biopsy and a conservative approach depends on the symptoms and the treatment options. The extent of surgical resection of spinal cord lesions depends largely on the intraoperative findings. In surgical cases, the final diagnosis depends on histological and molecular aspects. The radiologist plays an important role in the pretreatment diagnosis, providing support for the clinical and surgical decision-making process, in patients with spinal cord injury.
